# Inflammaging and Senescence-Driven Extracellular Matrix Remodeling in Age-Associated Cardiovascular Disease

**DOI:** 10.3390/biom15101452

**Published:** 2025-10-14

**Authors:** Ewelina Młynarska, Adrianna Kowalik, Agnieszka Krajewska, Natalia Krupińska, Weronika Marcinkowska, Jakub Motor, Aleksandra Przybylak, Katarzyna Tłustochowicz, Jacek Rysz, Beata Franczyk

**Affiliations:** 1 Department of Nephrocardiology, Medical University of Lodz, Ul. Zeromskiego 113, 90-549 Lodz, Poland; 2 Department of Nephrology, Hypertension and Family Medicine, Medical University of Lodz, Ul. Zeromskiego 113, 90-549 Lodz, Poland

**Keywords:** cardiovascular aging, inflammaging, cellular senescence, extracellular matrix remodeling, heart failure, atherosclerosis, RNA-based therapies, senolytics

## Abstract

Cardiovascular aging is a multifactorial and systemic process that contributes significantly to the global burden of cardiovascular disease, particularly in older populations. This review explores the molecular and cellular mechanisms underlying cardiovascular remodeling in age-related conditions such as hypertension, atrial fibrillation, atherosclerosis, and heart failure. Central to this process are chronic low-grade inflammation (inflammaging), oxidative stress, cellular senescence, and maladaptive extracellular matrix remodeling. These hallmarks of aging interact to impair endothelial function, promote fibrosis, and compromise cardiac and vascular integrity. Key molecular pathways—including the renin–angiotensin–aldosterone system, NF-κB, NLRP3 inflammasome, IL-6, and TGF-β signaling—contribute to the transdifferentiation of vascular cells, immune dysregulation, and progressive tissue stiffening. We also highlight the role of the senescence-associated secretory phenotype and mitochondrial dysfunction in perpetuating inflammatory and fibrotic cascades. Emerging molecular therapies offer promising strategies to reverse or halt maladaptive remodeling. These include senescence-targeting agents (senolytics), Nrf2 activators, RNA-based drugs, and ECM-modulating compounds such as MMP inhibitors. Additionally, statins and anti-inflammatory biologics (e.g., IL-1β inhibitors) exhibit pleiotropic effects that extend beyond traditional risk factor control. Understanding the molecular basis of remodeling is essential for guiding future research and improving outcomes in older adults at risk of CVD.

## 1. Introduction

Population aging is characterized by a growing proportion and absolute number of people aged 60 years and older, accompanied by a decline in the proportion and number of those aged 15 years or younger. Although first recognized in high-income countries, this demographic trend is now most evident in low- and middle-income regions, which currently host the majority of the world’s older population [1]. These changes are largely the result of major public health successes in controlling communicable diseases but simultaneously create the challenge of addressing the “double burden”—the coexistence of communicable and non-communicable diseases [2]. Globally, approximately 11% of the population is currently over the age of 60, and this figure is projected to rise to 22% by 2050 [3]. It is estimated that 23% of the global disease burden is attributable to conditions affecting those aged 60 years and older, with cardiovascular diseases accounting for the largest share (30.3%), followed by malignant neoplasms (15.1%), chronic respiratory diseases (9.5%), musculoskeletal conditions (7.5%), and neurological and psychiatric disorders (6.6%), including dementia, which has shown the most notable increase over the past decade and a half [4].

Disability-adjusted life years (DALYs) are driven primarily by ischemic heart disease (IHD), malignancies, chronic obstructive pulmonary disease (COPD), and cirrhosis in older men, whereas dementias, hearing loss, falls, hypertensive heart disease, musculoskeletal pain, and diarrheal diseases account for a greater share of health loss in older women. Mortality patterns also differ by sex: men exhibit higher death rates from most malignancies (except colorectal cancer), COPD, cirrhosis, and tuberculosis, whereas women experience higher mortality from cardiovascular disorders, dementias, and diarrheal diseases. IHD remains the leading cause of health loss and death across nearly all WHO regions, although infectious diseases still impose a substantial burden in Africa [5].

Metabolic syndrome, defined by central obesity, hypertension, dyslipidemia, and impaired glucose regulation, plays a central role in increasing the prevalence and lowering the age of onset of chronic diseases, including ischemic heart disease, among older individuals. Other metabolic disorders, such as cardiomyopathy and myocarditis, also contribute significantly to cardiovascular morbidity in the global population. Zhang and Cheng et al., analyzing Global Burden of Disease 2019 data, identified cardiomyopathy and myocarditis as common contributors to heart failure in aging populations. On a global scale, elevated systolic blood pressure and alcohol use rank among the most important risk factors for these cardiovascular outcomes [6].

Age itself constitutes a major independent determinant of cardiovascular disease (CVD) risk, but this effect is magnified by comorbidities such as obesity and diabetes, which exacerbate the age-related deterioration of cardiovascular health. Sex-specific differences further influence risk, as older women appear to be more susceptible to CVD than age-matched men. Nonetheless, in both sexes, cardiovascular risk escalates with age, a trend associated with the age-related decline of sex hormones, primarily estrogen and testosterone [7]. The progression of age-related cardiovascular dysfunction is reflected in rising rates of atherosclerosis, stroke, and myocardial infarction. According to the American Heart Association (AHA), the prevalence of CVD among U.S. adults is roughly 40% between ages 40 and 59, 75% between ages 60 and 79, and 86% in individuals over 80 [8,9,10].

A cross-sectional study in Salamanca, Spain, involving 480 individuals aged 65 years and older (327 interviewed; mean age 76; 64.5% female) found that 20.2% (95% CI: 15.8–24.5) had at least one cardiovascular disease. Ischemic heart disease was the most prevalent condition among men (12.1%; 95% CI: 6.1–18), whereas heart failure predominated among women (10.4%; 95% CI: 6.3–14.6). Hypertension emerged as the most common cardiovascular risk factor in both sexes (63.8% of men, 69.7% of women), followed by diabetes in men (36.2%) and a sedentary lifestyle in women (36.0%) [11].

Hallmarks of aging are defined as biological characteristics that not only emerge during the normal aging process and correlate with functional decline but also play a causative role in driving aging itself. Initially, nine hallmarks were recognized: genomic instability, telomere attrition, cellular senescence, epigenetic alterations, deregulated nutrient sensing, loss of proteostasis, mitochondrial dysfunction, stem cell exhaustion, and altered intercellular communication. In 2023, this framework was expanded to include three additional processes: impaired autophagy, chronic inflammation, and dysbiosis [12,13]. All of the above hallmarks are presented in Table 1.

**Figure 1:**
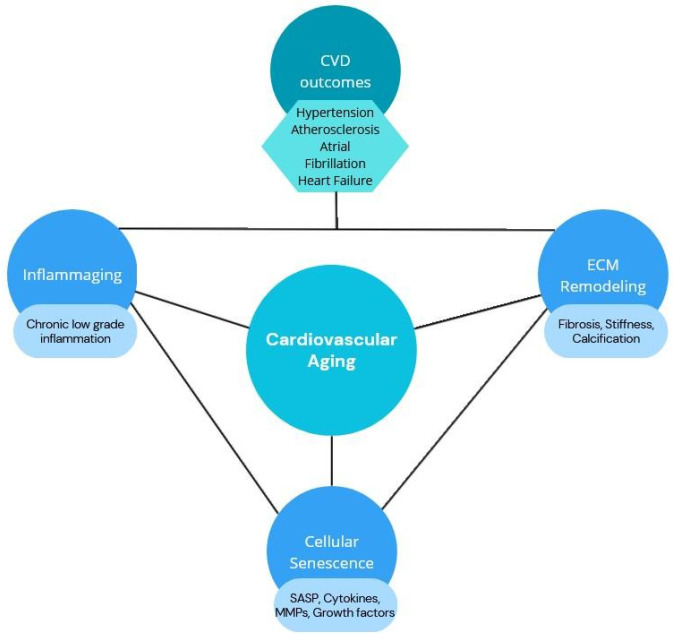
Schematic overview of molecular pathways involved in inflammaging [17,18].

Both inflammaging and cellular senescence function as interconnected causes and consequences of cardiovascular system aging (Figure 1). Chronic low-grade inflammation promotes tissue dysfunction, while senescent cells accumulate in the heart and vasculature, releasing pro-inflammatory factors that amplify damage. These processes have gained increasing attention as potential therapeutic targets. This work aims to present an up-to-date overview and highlight therapeutic strategies addressing their interplay.

## 2. Chronic Low-Grade Inflammation (Inflammaging) in Cardiovascular Aging

Chronic low-grade inflammation, or “inflammaging,” is a hallmark of aging and represents a distinct, systemic, and persistent inflammatory state that differs fundamentally from acute or general inflammation [17]. Unlike transient inflammatory responses to infection or injury, inflammaging arises without overt pathogenic triggers and is characterized by sustained, moderate elevations of pro-inflammatory mediators, including tumor necrosis factor alpha (TNF-α), interleukin 1 beta (IL-1β), IL-6, and C-reactive protein (CRP) [19]. Importantly, inflammaging is tightly linked to cellular senescence, mitochondrial dysfunction, and immune system remodeling, whereas general inflammation is typically a short-term pathogen- or damage-driven response [20]. Increasing evidence suggests that inflammaging plays a significant role in the development and progression of cardiovascular diseases, including myocardial infarction (MI) or coronary heart disease (CHD) [17,21].

The origins of inflammaging are multifactorial and involve variety mechanisms, leading to cellular senescence, including dysregulation of the gut microbiota, immune senescence, and mitochondrial dysfunction [22]. The gut microbiome, which plays a vital role in metabolic regulation and immune homeostasis, undergoes significant changes with age—marked by a loss of microbial diversity, reduced abundance of anti-inflammatory species, and an increase in pro-inflammatory pathobionts [23]. These changes are compounded by lifestyle factors common in old age, including polypharmacy, poor nutrition, and decreased physical activity [21]. Dysbiosis contributes to increased intestinal permeability, allowing microbial components such as lipopolysaccharide (LPS) to translocate into the bloodstream. This translocation stimulates Toll-like receptors (TLRs), especially TLR4 in endothelial and immune cells, thereby stimulating nuclear factor kappa-light-chain-enhancer of activated B cells (NF-κB) activation, leading to elevated production of pro-inflammatory cytokines and enhances T cell activation—directly implicating the aged microbiome in the pathophysiology of inflammaging [21,24,25].

**Figure 2:**
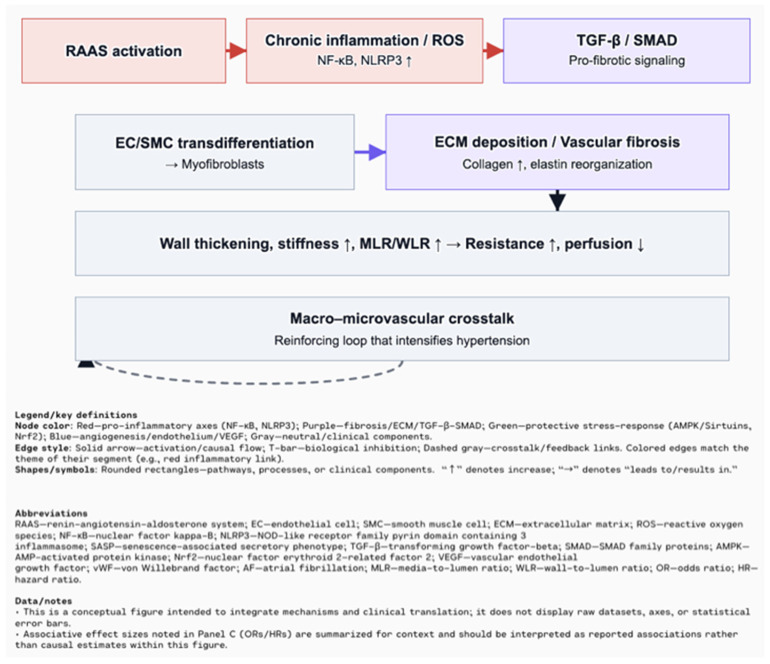
Hypertension and vascular remodeling: RAAS activation and chronic inflammation/ROS (NF-κB, NLRP3) drive TGF-β/SMAD signaling, EC/SMC transdifferentiation to myofibroblasts, and ECM deposition (collagen ↑, elastin reorganization). Consequences include wall thickening, stiffness ↑, MLR/WLR ↑ → resistance ↑ and perfusion ↓, with macro–microvascular crosstalk reinforcing hypertension [17,22].

The persistent release of pro-inflammatory cytokines triggers pro-inflammatory signaling in vascular and cardiac tissue, impairing endothelial function and promoting ECM remodeling. In this process, the transcription factor NF-κB plays a key role, regulating the expression of pro-inflammatory cytokines, adhesion molecules, and chemokines, thereby facilitating leukocyte adhesion and inducing vascular inflammation [17]. A well-recognized initiator of the NF-κB-mediated inflammatory responses is the activation of the Nod-like receptor pyrin domain-containing 3 (NLRP3) inflammasome [21]. Composed of the sensor protein NLRP3, the adaptor ASC, and pro-caspase-1, this multi-protein complex becomes activated in response to various cellular stress signals. The activation process involves two main stages: priming, which is initiated by endogenous and exogenous stressors that trigger NF-κB-mediated upregulation of NLRP3, and triggering, which leads to inflammasome assembly and subsequent activation of caspase-1. Activated caspase-1 cleaves pro-IL-1β and pro-IL-18 into their mature, pro-inflammatory forms. Studies in aged animals demonstrate elevated expression of IL-1β and IL-18, with the absence of NLRP3 or ASC significantly reducing these cytokines, providing strong evidence for the inflammasome’s involvement in age-related chronic inflammation [22]. Chronic activation of NF-κB and the NLRP3 inflammasome contributes not only to endothelial dysfunction but also to myocardial fibrosis and remodeling in age-related cardiovascular disease (Figure 2). Remodeling is a dynamic and often reversible process of ECM reorganization that can be both physiological and pathological, whereas fibrosis is a maladaptive, irreversible outcome of remodeling characterized by excessive ECM deposition and tissue scarring. This persistent inflammatory state promotes the development of heart failure with preserved ejection fraction (HFpEF), which is highly prevalent in older adults [21,26].

**Figure 3:**
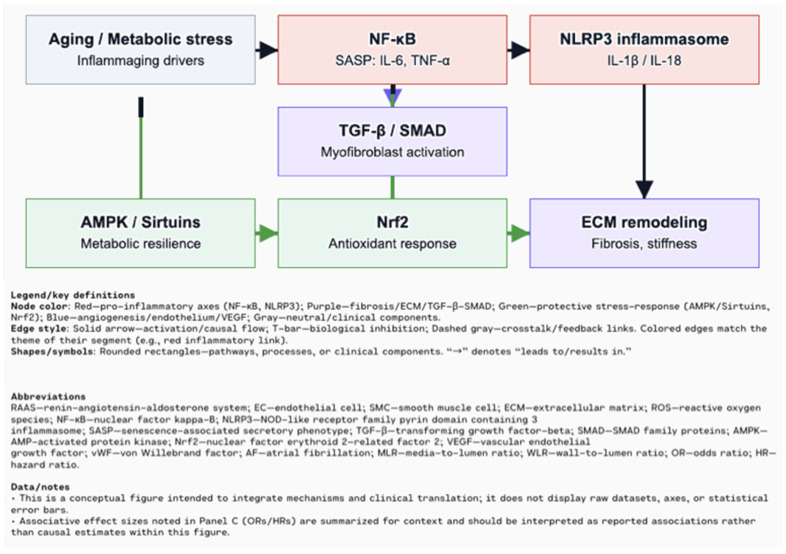
Inflammaging and cellular senescence: aging/metabolic stress fosters inflammaging via NF-κB (SASP: IL-6, TNF-α) and NLRP3 (IL-1β/IL-18), which promote TGF-β/SMAD and ECM remodeling. Protective stress-response axes (AMPK/Sirtuins; Nrf2) inhibit upstream inflammatory drivers and support metabolic/antioxidant resilience [21].

**Figure 4:**
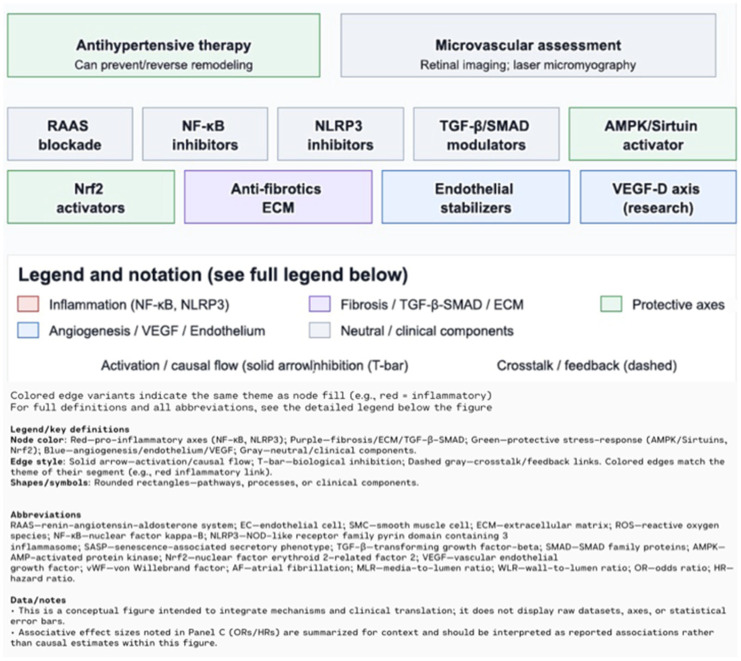
Clinical translation and targets: antihypertensive therapy can prevent or reverse adverse remodeling. Microvascular assessment (e.g., retinal imaging; laser micromyography) complements risk evaluation. Therapeutic levers include RAAS blockade; NF-κB/NLRP3 inhibitors; TGF-β/SMAD modulators; AMPK/Sirtuin and Nrf2 activators; anti-fibrotics/ECM strategies; and endothelial stabilizers (including research on the VEGF-D axis) [17].

Importantly, NLRP3 inflammasome activation emerges as a crucial mechanism linking mitochondrial dysfunction with inflammation in aging vasculature. Mitochondrial damage and reactive oxygen species (ROS) overproduction result in the release of mitochondrial DNA (mtDNA) into the cytosol, where it acts as a damage-associated molecular pattern (DAMP), triggering NLRP3 activation. This, in turn, amplifies the production of IL-1β and IL-18—cytokines strongly associated with vascular inflammation, atherosclerosis, and plaque instability (Figure 3). Interestingly, studies highlight that IL-18 expression increases with age, particularly in the female heart, pointing to possible sex-specific inflammatory patterns in cardiovascular ageing. Moreover, inflammageing-induced oxidative stress impairs endothelial nitric oxide (NO) bioavailability, increases vascular permeability, and promotes low-density lipoprotein (LDL) oxidation—key early events in atherogenesis. Activated monocytes and macrophages further aggravate this state by producing ROS and transforming into foam cells, which contribute to plaque formation and vascular stiffening, both hallmarks of arterial aging. Inflammatory biomarkers such as high-sensitivity C-reactive protein (hsCRP), IL-6, and IL-1β serve as indicators of this ongoing process and are closely linked to increased cardiovascular risk [17]. Adding to this process, the age-related decline in 5’ AMP-activated protein kinas (AMPK) and sirtuins (Sirt1, Sirt3) undermines mitochondrial quality control and promotes inflammatory signaling. AMPK, a key energy sensor, normally sustains mitochondrial homeostasis and inhibits NF-κB, while Sirt1, through NAD^+^-dependent mechanisms, supports AMPK and represses pro-inflammatory gene expression (Figure 4). With ageing, both AMPK activation and sirtuin levels decrease—due to NAD^+^ depletion and increased CD38 activity—leading to the accumulation of dysfunctional mitochondria, elevated ROS, and mtDNA release. These changes reinforce NLRP3 inflammasome activation, establishing a vicious cycle between metabolic dysfunction and vascular inflammation in the ageing heart [21]. Counterbalancing these pro-inflammatory processes is the nuclear factor erythroid 2–related factor 2 (Nrf2) pathway. As a master regulator of the cellular antioxidant response, Nrf2 orchestrates the transcription of genes involved in ROS detoxification, mitochondrial protection, and inflammation resolution. Under physiological conditions, Nrf2 activity declines with age, contributing to impaired redox homeostasis and higher oxidative burden. This reduction exacerbates mitochondrial vulnerability, particularly by allowing ROS accumulation and limiting the upregulation of protective enzymes [27].

## 3. Cellular Senescence in the Cardiovascular System

Cardiomyocyte senescence is associated with multiple functional impairments, including activation of the DNA damage response (DDR), endoplasmic reticulum (ER) stress, mitochondrial dysfunction, contractile abnormalities, hypertrophic remodeling, and expression of the senescence-associated secretory phenotype (SASP) [28].

Cellular senescence plays a fundamental role in the biological mechanisms driving aging [29]. It is a heterogeneous and context-dependent cell fate characterized by irreversible cell cycle arrest, with phenotypic features varying by cell type and environmental context [30]. Senescent cells permanently exit the cell cycle and exhibit resistance to apoptosis, enhanced protein synthesis, metabolic reprogramming—including increased glycolytic activity, reduced fatty acid oxidation, and elevated ROS production and develop the SASP [31]. Beyond aging and pathology, senescent cells participate in physiological processes such as wound healing, tissue remodeling, fibrosis, and embryonic development. Under tightly regulated conditions, their presence may support tissue homeostasis and regeneration [32,33,34]. The SASP critically modulates the tissue microenvironment by promoting the recruitment, retention, and activation of immune cells, which mediate senescent cell clearance [29]. SASP components include pro-inflammatory cytokines, bradykinins, prostanoids, microRNAs, DAMPs, chemokines, and factors such as activin A that impair stem cell function. It also encompasses hemostatic regulators (e.g., PAI-1, PAI-2), vasoactive mediators, and proteolytic enzymes such as matrix metalloproteinases that degrade the extracellular matrix [31,35] (Table 2).

Metabolic homeostasis is essential for cardiomyocyte function. With aging and stress, metabolic shifts occur that promote dysfunction and senescence. Non-myocyte cells within the cardiac microenvironment—including endothelial cells, fibroblasts, vascular smooth muscle cells (VSMCs), and immune cells (macrophages, neutrophils, T and B lymphocytes, NK cells), as well as resident progenitor and mesenchymal stromal cells, contribute to this process via the modulation of inflammation, fibrosis, vascular tone, and extracellular matrix (ECM) remodeling. Senescent cardiomyocytes, in turn, influence the microenvironment, promoting either adaptive or maladaptive remodeling [28].

Endothelial cells, due to their constant exposure to circulating blood, shear stress, and pathogenic stimuli, are highly susceptible to senescence. Tissues with dense vascularization thus show greater senescent cell accumulation [36]. Senescent endothelial cells are found in atherosclerosis, HFpEF, and atrial fibrillation, suggesting their involvement in vascular and myocardial remodeling [37]. Mechanisms driving endothelial senescence include dysregulated NO and endothelium-derived hyperpolarizing factor signaling, altered calcium homeostasis, increased permeability, impaired angiogenesis, and reduced mitochondrial biogenesis. These are aggravated by oxidative stress, cell cycle disruption, vascular inflammation, and metabolic disorders like hyperuricemia. Key molecular pathways involved include Sirt1, Klotho, FGF21, and the renin–angiotensin–aldosterone system (RAAS), along with accumulated genetic and epigenetic alterations [38].

Cardiac fibroblasts are central to ECM remodeling and paracrine communication within the heart [39]. Following myocardial infarction, activation of the p53/p21 pathway induces their senescence, which may limit fibrosis via SASP proteins such as CCN1 [40,41]. Age-related metabolic disturbances, including mitochondrial dysfunction and defective mitophagy, promote cardiomyocyte senescence by increasing ROS production and oxidative stress [42,43]. Activation of p53 and regulators such as Rb1 and Meis2 further exacerbate mitochondrial and metabolic dysfunction [44,45].

Senescent cardiomyocytes adopt a SASP driving inflammation and fibrotic remodeling through fibroblast activation [41,46]. Epigenetic changes and dysregulation of miRNAs (e.g., miR-34, miR-17-92) contribute to impaired contractility and fibrosis [47,48].

Senescent VSMCs contribute significantly to atherosclerosis and pulmonary hypertension [37]. Their senescence is driven by telomere attrition, DNA damage, oxidative stress and the SASP of these cells promotes immune cell recruitment and endothelial activation through the secretion of pro-inflammatory cytokines [49,50,51]. These cells reduce collagen synthesis, increase elastase and matrix-degrading protease secretion, and shift toward an osteogenic phenotype, leading to arterial stiffening and calcification [49,52,53]. Stifness refers to the increased rigidity of tissue mainly due to excessive collagen deposition and cross-linking, whereas calcification is the pathological deposition of calcium salts within the tissue, leading to hardening through mineralization.

Immune cells exert dual roles in regulating cardiomyocyte senescence and cardiac remodeling [28]. Macrophages mediate clearance of senescent cells but also promote senescence via NLRP3 inflammasome activation and IL-1β secretion, contributing to electrophysiological alterations and calcium dysregulation in diabetic hearts [54,55]. Inflammatory CCR2^+^ macrophages can also support regeneration via IL-4/IL-13 signaling [56]. Macrophage-mediated inflammatory signaling contributes to cardiomyocyte hypertrophy [57]. Neutrophils may exacerbate post-MI injury and senescence [58]. Mast cells preserve cardiac function after MI via tryptase-mediated protein kinase A regulation, but, in diabetes, they promote fibrosis and apoptosis [59,60]. T cells also affect cardiomyocyte fate: δT lymphocytes predominantly produce IL-17A and it promotes apoptosis during ischemia/reperfusion, while regulatory T cells protect against Ang II-induced fibrosis and release paracrine factors (Cystatin F, TNFSF11, IL-33, FGL-2, matrilin-2, IGF-2) that foster regeneration and suppress senescence after MI [58,61,62].

## 4. Extracellular Matrix Remodeling Mediated by Inflammation and Senescence

The ECM is a complex and dynamic network composed of proteins, proteoglycans, polysaccharides, and biologically active factors [64]. It plays a crucial role in maintaining tissue integrity and function by undergoing remodeling in response to inflammation or injury, adapting its structure and composition to maintain tissue integrity and function. However, a persistent expansion of the ECM may evolve into maladaptive fibrosis and organ dysfunction. This pathological remodeling can be triggered by various factors such as hypoxia, inflammation, biomechanical stress, and excessive neurohormonal activation [65].

Inflammation contributes to ECM remodeling by releasing cytokines that activate fibroblasts, increasing the production of ECM components. It also upregulates matrix metalloproteinases (MMPs) that degrade ECM proteins. This dual action can lead to pathological ECM remodeling, contributing to fibrosis and tissue dysfunction. Senescence, on the other hand, leads to the accumulation of senescent cells that secrete pro-inflammatory factors known as the SASP. SASP factors, including cytokines, chemokines, growth factors, and proteases, further alter the ECM by promoting degradation, impairing its turnover, and reshaping its composition [66]. For example, senescent hepatic stellate cells or fibroblasts upregulate multiple MMPs while frequently downregulating their inhibitors, tissue inhibitors of metalloproteinases (TIMPs), thereby shifting the MMP/TIMP balance toward excessive proteolysis. At the same time, other SASP components such as ADAMs and ADAMTSs promote ECM cleavage and growth factor release, amplifying tissue damage. Conversely, ECM components including collagen I or fibronectin can themselves influence entry into senescence, demonstrating a bidirectional feedback loop between ECM state and cellular senescence. ECM is categorized into two primary types based on location: the interstitial connective tissue matrix and the basement membrane [67]. The interstitial matrix surrounds cells and is primarily composed of fibrillar proteins including collagen, fibronectin and elastin [68]. Its main function is to form a meshwork that connects structural cell types within tissues. The basement membrane includes components such as collagen IV laminin, fibronectin, and hyaluronan, produced by epithelial and endothelial cells. Its primary physiological functions include separating epithelial cells from the surrounding matrix and regulating cell differentiation [68]. Excessive accumulation of ECM components, particularly fibronectin, collagen, and proteoglycans, plays a crucial role in the pathogenesis and progression of fibrosis and organ dysfunction [67]. Abnormal deposition of ECM components is evident in clinical conditions such as lung fibrosis, liver cirrhosis, and cardiovascular diseases [69].The ECM serves a dual role in fibrosis, acting as both an indicator and an active agent in disease progression. ECM contributes to disease advancement via the accumulation of its components and by creating a positive feedback loop in fibrotic disease. The specific ECM components deposited vary depending on the type of fibrotic disease. For example, in solid organs such as the kidney and liver, the deposition of elastin in the walls can disrupt blood supply and lead to organ dysfunction. In cardiac fibrosis, the accumulation of collagen isoforms in the ventricular wall reduces wall compliance and lowers heart ejection capacity [70].

The positive feedback loop in fibrotic diseases operates through a series of interconnected mechanisms that drive disease progression. At its core, this cycle involves ECM deposition, leading to increased matrix stiffness, which triggers cellular responses that ultimately promote further ECM accumulation. The process begins with ECM deposition in the tissue microenvironment, creating a stiffer mechanical environment. This increased stiffness acts as a signal that activates enzymatic processes involving lysyl oxidase (LOX) and LOX-like (LOXL) enzymes [71].These enzymes catalyze the formation of aldehydes from specific amino acids (lysine residues) found in collagen and elastin proteins, forming strong cross-links between collagen fibers, which significantly increases the mechanical stiffness of the tissue matrix. The increased matrix stiffness has two major consequences: it inhibits natural ECM degradation processes, allowing more ECM to accumulate, and it creates enhanced mechanical stress signals that activate downstream signaling pathways. These activated pathways trigger several cellular responses: they promote epithelial–mesenchymal transition (EMT), they activate ECM-producing myofibroblasts, and they lead to miR-29 downregulation, which removes a crucial negative regulatory mechanism [72]. This entire sequence creates a self-reinforcing cycle where ECM deposition leads to increased stiffness, which triggers responses that promote more ECM production. As tissue fibrosis progresses, this cycle continues to amplify, leading to excessive ECM abundance in the tissue microenvironment and driving disease progression forward [73].

A study investigated the factors contributing to increased tissue stiffness in idiopathic pulmonary fibrosis (IPF) by examining collagen content and post-translational modifications. The study identified differences in the expression of collagen cross-linking enzymes in IPF tissue. It showed that, while messenger RNA (mRNA) levels of LOX and LOXL1 remained unchanged, there were significant increases in the expression of LOXL2, LOXL3, and LOXL4. Additionally, no correlation was found between lung tissue stiffness and collagen concentration. This was accompanied by increased amine oxidase activity in IPF lung tissue sections. The findings suggest that post-translational modifications, particularly increased cross-linking enzyme activity, rather than collagen amount, contribute to the increased stiffness observed in IPF tissues [74].

MMPs are a family of endopeptidases involved in the degradation of ECM components, including collagen, fibronectin, laminin, and gelatin [75]. MMPs are produced by various cell types, including fibroblasts, osteoblasts, endothelial cells, vascular smooth muscle cells, immune cells such as macrophages and lymphocytes, and placental cells. Under normal conditions, MMP activity is low.

During inflammation or tissue repair, their activity significantly increases. MMPs contain several functional domains, with the catalytic domain featuring a conserved Zn^2+^ binding motif that enables the enzyme to break down peptide bonds [76]. Various factors, such as inflammatory cytokines, growth factors, oxidative stress, or mechanical stress, can upregulate MMPs expression and promote their activation during processes including wound healing, inflammation, and tissue repair [77].

Tissue inhibitors of metalloproteinases (TIMPs) are endogenous MMP inhibitors. The N-terminal end of TIMP folds inward and wedges into the active site of MMPs, inhibiting their hydrolytic activity. This binding is facilitated by cysteine residues in the N-terminal domain that chelate the zinc ion in the MMPs active site, effectively blocking the enzyme’s catalytic activity [78]. A well-controlled balance between the function of MMPs and TIMPs is critical in maintaining ECM homeostasis. The MMP/TIMP concentration ratio influences proteolysis efficacy, with implications for angiogenesis, inflammation, tissue regeneration, and fibrogenesis.

A study by Bjørnstad JL et al. examined the relationship between MMPs and myocardial remodeling in patients with severe aortic stenosis (AS) [79]. They analyzed circulating levels of MMPs before and after aortic valve replacement surgery. Key findings included stable MMP-2 levels, significant increases in MMP-3 and MMP-9 two days post-operatively, and correlations between MMP-3 and markers of cardiac stress or remodeling [79]. Several MMPs and TIMPs may be involved in the vascular remodeling associated with hypertension. Some studies have shown a correlation between MMP levels and hypertension. Onal et al. conducted a study in which they compared serum levels of MMP-9 and TIMP-1 in patients with hypertension versus normotensive individuals [80]. Key findings included higher pre-treatment MMP-9 levels and lower TIMP-1 levels in hypertensives, which normalized after three months of antihypertensive therapy. An increased MMP-9 activity could lead to excessive elastin degradation, while decreased TIMP-1 may result in abnormal collagen deposition. These findings suggest MMPs play a role in hypertensive target organ disease pathogenesis [80].

Macrophages, through a secretion of MMPs, play a crucial role in ECM degradation during fibrosis [67]. They also ingest and digest ECM components via integrin-mediated phagocytosis and receptor-mediated endocytosis. The interaction between macrophages, MMPs, and the ECM forms a key feedback loop in fibrogenesis, with changes in ECM composition affecting macrophage function. Products of ECM degradation, termed matricryptins or matrikines, can regulate macrophage behavior, influencing their phenotype and chemotaxis [81]. This complex interplay between macrophages, MMPs, and the ECM is essential for understanding and potentially targeting fibrotic processes.

The relationship between ECM remodeling and endothelial dysfunction represents a complex pathophysiological process that plays a crucial role in vascular disease progression [82]. This interaction involves dynamic, bidirectional communication between VSMCs and the ECM, which serves as a critical structural component of the vessel wall, primarily produced by VSMCs. Three primary ECM types—elastic fibers, fibrillar collagen, and large aggregating proteoglycans—maintain the mechano-elastic properties of arteries. ECM content is determined by the delicate balance between production and degradation, with MMPs facilitating VSMC migration by degrading the surrounding connective tissue cage [83]. Importantly, aging disrupts the endothelial balance between vasodilators like NO and vasoconstrictors, leading to oxidative stress, reduced NO bioavailability, and mitochondrial dysfunction. These alterations not only impair vascular tone but also promote maladaptive ECM remodeling through enhanced collagen deposition, elastin degradation, and MMP activation. Thus, endothelial dysfunction and ECM remodeling reinforce each other in a vicious cycle that drives arterial stiffening and calcification [84].

## 5. Clinical Implications in Major Cardiovascular Diseases

**Figure 5:**
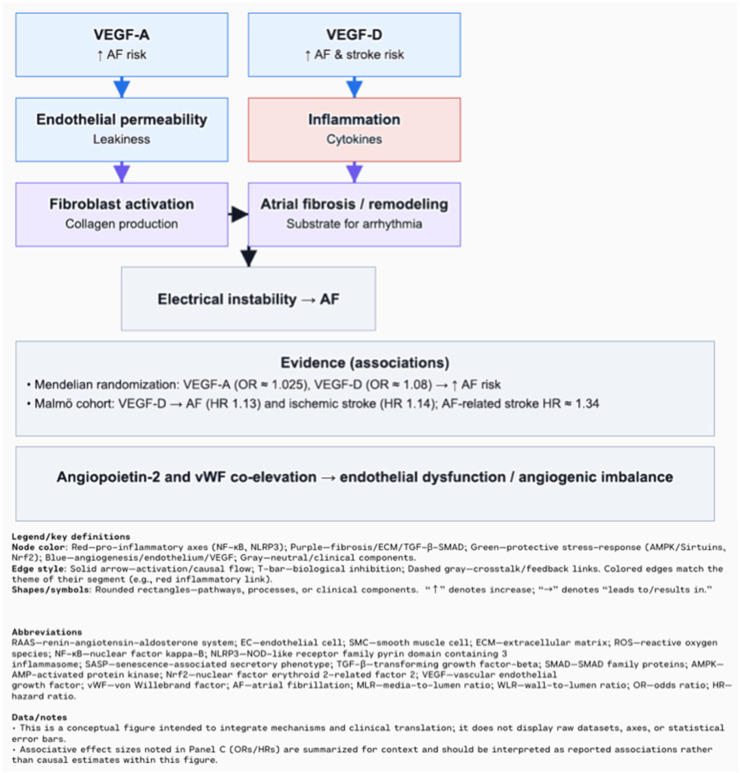
VEGF and AF: ↑ VEGF-A and ↑ VEGF-D → ↑ AF risk (and stroke for VEGF-D), plausibly via increased endothelial permeability and inflammation → fibroblast activation → atrial fibrosis/remodeling → electrical instability → AF. Supporting associations are summarized in the evidence box [93,94]. Explanation of the symbols: “Arrows indicate causal relationships (“→” = leads to; “↑” = increase)”.

In hypertension, structural changes in the vasculature are common, affecting both small resistance arteries and large conduit arteries. These changes lead to an increased media-to-lumen ratio (MLR) or wall-to-lumen ratio (WLR), which impairs blood flow and increases vascular resistance [85]. Sustained activation of the RAAS, chronic inflammation, and oxidative stress contribute to the transdifferentiation of endothelial and smooth muscle cells into myofibroblasts, resulting in excessive ECM deposition and permanent vascular fibrosis [86]. This fibrosis is characterized by vessel wall thickening, loss of elasticity (vascular stiffness), and ECM reorganization, all of which further increase resistance and impair tissue perfusion [87,88]. Crosstalk between structural changes in large and small vessels forms a feedback loop that intensifies hypertension and drives pathological remodeling in both the macro- and microcirculation [85,88]. Appropriate antihypertensive therapy can prevent, or in some cases even reverse, these vascular changes. Emerging non-invasive techniques for assessing microvascular structure, such as retinal imaging or laser-based micromyography, offer promising tools for clinical implementation [85]. A systematic review of eight studies showed that patients with atrial fibrillation (AF) often have elevated levels of vascular endothelial growth factor (VEGF), particularly VEGF-A and VEGF-D, which demonstrated stronger associations with AF than VEGF-C [89]. VEGF contributes to AF pathogenesis through atrial remodeling, inflammation, increased vascular permeability, and stimulation of fibrosis, ultimately leading to electrical instability in atrial tissue [89,90]. Mendelian randomization analysis confirmed a causal association: genetically predicted higher levels of VEGF-A (OR ≈ 1.025, PFDR ≈ 0.06) and VEGF-D (OR ≈ 1.08, PFDR = 0.001) were significantly linked to increased AF risk, whereas VEGF-C and VEGF receptors (VEGFR-2/3) showed no such associations [91]. VEGF-D was also associated with atrial flutter (OR ≈ 1.071, PFDR ≈ 0.087), and sensitivity analyses confirmed the robustness of the VEGF-D–AF association, supporting its potential causative role [91]. In the Malmö Diet and Cancer study, higher circulating VEGF-D was associated with increased risk of both AF (HR 1.13) and ischemic stroke (HR 1.14), with stroke risk being higher in AF patients (HR for AF-related stroke = 1.34) [92]. Mechanistically, VEGF-D may promote atrial fibrosis by stimulating fibroblast migration and collagen production, thereby creating a substrate for sustained arrhythmias [91]. Moreover, co-elevation of angiopoietin-2 and von Willebrand factor suggests a link between endothelial dysfunction and angiogenic imbalance in AF patients [93,94] (Figure 5).

Atherosclerosis is a chronic inflammatory disease of the arteries, where plaque formation and progression are strongly linked to immune responses and inflammation. As plaques enlarge, their composition changes—the fibrous cap thins, and the lipid core expands, leading to features of vulnerable plaques with increased risk of rupture and acute coronary syndromes. These processes are driven by inflammatory cell infiltration (macrophages, T lymphocytes) and the secretion of cytokines and proteolytic enzymes such as MMPs that degrade collagen in the cap [95]. Another destabilizing factor is the presence of microhemorrhages and neovascularization within plaques. These fragile, leaky vessels increase the risk of intraplaque bleeding and further core expansion, accelerating plaque progression and rupture [96]. Endothelial cell senescence also plays a crucial role. As shown in the article “New Dawn for Atherosclerosis”, aging endothelial cells lose their repair function and adopt a SASP, releasing pro-inflammatory cytokines and matrix-degrading enzymes, thus promoting chronic inflammation and plaque instability [97]. Imaging and histopathological data confirm that thin-cap fibroatheromas (TCFA)—plaques with fibrous caps < 65 μm, large lipid cores, and inflammation—are major causes of thrombosis leading to myocardial infarction. These plaques are hard to detect non-invasively, prompting the development of advanced imaging techniques to better identify and assess their risk [97,98]. Emerging imaging modalities, such as advanced ultrasound and MRI, are increasingly capable of detecting features of plaque vulnerability, including structural integrity loss, microfissures, and inflammatory activity. These tools are becoming essential for cardiovascular risk assessment [99].

Heart failure (HF) is a complex clinical syndrome resulting from the heart muscle’s insufficient ability to effectively pump blood, leading to tissue hypoxia and severe hemodynamic disturbances. A key element in the pathophysiology of HF is the loss of cardiomyocytes—specialized cardiac muscle cells responsible for myocardial contractility. In the adult heart, cardiomyocytes have a very limited capacity for proliferation and regeneration, meaning that damaged or dead cells are not effectively replaced by new ones. As a result, following events such as acute myocardial infarction or chronic cardiac overload, cardiomyocyte loss becomes irreversible, promoting replacement fibrosis. This process promotes the development of so-called replacement fibrosis, where the cardiac muscle is replaced by fibrotic tissue, significantly impairing its mechanical function and electrical impulse [100]. The process of cardiac fibrosis is closely related to the activation of fibroblasts, which under physiological conditions are responsible for maintaining the structure of the ECM and its remodeling. In pathological conditions, following heart injury, fibroblasts become activated and differentiate into myofibroblasts—cells capable of producing large amounts of ECM components, such as type I and III collagen, fibronectin, and proteoglycans. Excessive ECM deposition increases the stiffness of the heart walls, leading to reduced compliance (so-called diastolic dysfunction) and impaired contractile efficiency (systolic dysfunction). This exacerbates heart failure and increases the risk of arrhythmias and sudden cardiac death [101]. At the molecular level, fibroblast activation is induced by various signals, including pro-inflammatory cytokines such as TGF-β, TNF-α, and IL-6. TGF-β is considered the main mediator inducing the transition of fibroblasts into myofibroblasts by stimulating their proliferation, migration, and production of collagen and other ECM components. Furthermore, cytokines including TNF-α can act through autocrine and paracrine mechanisms, intensifying inflammation and further activating fibroblasts. Interactions between fibroblasts and immune cells, mainly macrophages, form a complex signaling network that regulates cardiac remodeling and is crucial for the transition from acute to chronic heart failure [102].

Advanced molecular biology techniques, such as single-cell RNA sequencing (scRNA-seq), are increasingly important in HF research. These methods allow the identification of different fibroblast populations present in the heart, which vary in gene expression profiles and function. In heart failure, fibroblasts involved in fibrogenesis differ between HFpEF and HFrEF (heart failure with reduced ejection fraction). In HFpEF, fibroblasts are mainly activated by metabolic stress and hypoxia signaling, whereas, in HFrEF, myofibroblasts and so-called matrifibrocytes that produce ECM predominate. This detailed characterization enables the identification of new therapeutic targets and potential diagnostic markers [103]. Recent years have also revealed epigenetic mechanisms regulating fibroblast activation. For example, the enzyme ATP-citrate lyase (ACLY), involved in cellular metabolism, appears to be critical for maintaining the active gene program of myofibroblasts. Inhibition of ACLY in animal models leads to fibroblast deactivation and reduced fibrosis, opening up new prospects for therapies aimed at blocking fibrogenesis in heart failure [100]. In summary, heart failure results from both cardiomyocyte loss and pathological fibroblast activation, which lead to fibrosis and permanent remodeling of the heart muscle. Understanding the molecular mechanisms of these processes is crucial for developing new therapies aimed at halting disease progression and improving the quality of life for patients with heart failure [100].

## 6. Sex Differences in Cardiovascular Aging and the Impact of Comorbidities

Experimental and clinical evidence consistently demonstrates that biological sex is a major determinant of cardiovascular aging. In premenopausal women, estrogen—particularly signaling via ERβ—exerts protective effects by suppressing fibrosis, apoptosis, and excessive activation of proinflammatory and oxidative stress pathways [104]. Estrogen also preserves mitochondrial function, downregulates ECM gene expression, inhibits TGF-β signaling, and reduces MMP activity, collectively limiting maladaptive remodeling of the myocardium and vasculature. A Prospective cohort study and meta-analysis showed that higher circulating 17β-estradiol (E2) levels are associated with lower cardiovascular risk, whereas earlier menopause increases susceptibility to heart failure, underscoring the cardioprotective role of estrogen [105].

Following menopause, the loss of estrogen signaling accelerates vascular stiffening, promotes accumulation of fibrillar collagens (types I and III), enhances LOX–mediated collagen crosslinking, and amplifies vascular inflammation, together driving an accelerated trajectory of vascular aging and heightened risk of heart failure in older women [106]. In contrast, experimental data indicate that higher testosterone levels in males may accelerate vascular fibrosis and adverse remodeling via TGF-β activation, increased oxidative stress, and enhanced apoptosis of endothelial and smooth muscle cells [107].

A Recent large-scale systematic review and meta-analysis, encompassing 167 studies and 509,743 participants, confirmed that men exhibit higher arterial stiffness, as measured by pulse wave velocity (PWV), at younger ages than women, suggesting an earlier onset of vascular remodeling in males. After menopause, women experience a rapid increase in PWV, reflecting the loss of estrogen-mediated protection and partially narrowing the sex gap in older age [108]. Moreover, comorbidities such as diabetes and obesity synergistically accelerate vascular aging through complementary molecular mechanisms. In obesity, adipose-tissue-derived inflammatory cytokines and metabolic stress activate pro-fibrotic TGF-β/SMAD signaling, enhance fibrillar collagen I and III deposition, and impair ECM turnover, thereby driving medial fibrosis and vascular stiffening [109]. In parallel, reduced levels of adiponectin—a key anti-inflammatory and anti-fibrotic adipokine—remove inhibitory constraints on fibroblast activation and α-SMA/collagen expression, amplifying vascular fibrosis [110]. Diabetes further aggravates these processes by inducing chronic hyperglycemia-driven formation of advanced glycation end-products (AGEs), which crosslink collagen fibers and activate the RAGE–NF-κB pathway, perpetuating vascular inflammation and oxidative injury [111]. In addition, insulin resistance and impaired PI3K/Akt/eNOS signaling reduce NO bioavailability, exacerbating endothelial dysfunction and impairing vascular repair [112]. These findings underscore the necessity of integrating sex as a fundamental biological variable, together with comorbidities such as diabetes and obesity, into research on vascular aging and the design of precision cardiovascular interventions. 

## 7. Therapeutic Opportunities and Future Directions

Processes associated with inflammaging and senescence-driven extracellular matrix remodeling represent promising therapeutic targets for the prevention and treatment of age-associated cardiovascular diseases.

Statins have long been used in the prevention of coronary artery disease. Their main mechanism involves lowering serum cholesterol by inhibiting hepatic cholesterol synthesis, which leads to the upregulation of hepatic low-density lipoprotein (LDL) receptors and enhanced clearance of LDL-cholesterol. In addition to their lipid-lowering properties, statins may also confer cardiovascular protection through LDL-independent mechanisms, known as pleiotropic effects [113].

The pleiotropic effects of statins have long been recognized, particularly their ability to reduce CRP levels, highlighting their anti-inflammatory properties beyond lipid lowering. According to the JUPITER study by Ridker P. et al., 17,802 apparently healthy men and women with LDL cholesterol levels below 130 mg/dL and high-sensitivity CRP levels of 2 mg/L or higher were randomly assigned to receive either rosuvastatin 20 mg daily or placebo [114]. The study showed that rosuvastatin reduced LDL-C by 50%, CRP by 37%, and the primary end point by 44% [114]. The analysis of the JUPITER trial suggests that the clinical benefits of rosuvastatin were greater than expected based on LDL-C reduction alone, indicating that additional, non–lipid-lowering (pleiotropic) effects may contribute to its overall efficacy [113].

According to a meta-analysis by Wang J. et al., which included 25 eligible studies comprising 7921 participants with chronic kidney disease (CKD), statin therapy was significantly associated with a reduction in CRP levels (mean difference: −2.06 mg/L) [115]. Subgroup analyses, sensitivity tests, and trim-and-fill methods confirmed the robustness and stability of these pooled results [115]. However, further research is needed to evaluate these anti-inflammatory effects specifically in patients with cardiovascular diseases.

Statins exert pleiotropic effects beyond their lipid-lowering properties, significantly impacting cardiovascular and inflammatory diseases. By inhibiting isoprenoid synthesis, they disrupt the posttranslational modification of small GTP-binding proteins such as Rho, Rac and ROCK, which leads to attenuation of pro-inflammatory and pro-thrombotic signaling pathways. These effects result in increased endothelial NO synthase (eNOS) activity, reduced oxidative stress, inflammatory cell infiltration, cytokine production, and platelet aggregation. Consequently, statins improve endothelial function, reduce vascular inflammation and cardiac hypertrophy, and enhance myocardial perfusion, contributing to their overall therapeutic benefit [116].

Moreover, upregulation of Nrf2 activity may offer therapeutic benefits in conditions such as diabetic cardiomyopathy and mitochondrial dysfunction, and in mitigating age-related changes in the heart [117]. Nuclear factor erythroid 2-like 2 (NFE2L2; commonly known as Nrf2 has been identified as a major regulator of the oxidant/antioxidant balance [117]. The Nrf2 pathway plays a key role in antioxidant defense and is closely associated with oxidative-stress-induced cardiac remodeling [118].

Wang et al. studied the effects of the proteasome inhibitor MG-132 on diabetic cardiomyopathy in OVE26 type 1 diabetic mice. Treating three-month-old mice with MG-132 (10 μg/kg/day) for three months improved cardiac function, reversed remodeling, and reduced oxidative stress and inflammation [119]. These benefits were linked to increased Nrf2 activity and decreased NF-κB signaling. The results indicate that MG-132 may be a promising therapy for diabetic cardiomyopathy by restoring redox balance and reducing inflammation [119].

Further preclinical and clinical studies are required to develop effective drugs targeting the Nrf2 pathway for the treatment of atherosclerosis and other cardiovascular diseases. However, clinical application requires caution due to issues with bioavailability, potential toxicity, and tumorigenic risks associated with sustained activation [120].

IL-6, initially known as a pro-inflammatory cytokine, is now recognized as a pleiotropic, hormone-like mediator involved in immune regulation, metabolism, and endothelial and neuronal function, with expression induced mainly by upstream cytokines TNF-α and IL-1β. The CANTOS trial showed that canakinumab, an IL-1β inhibitor, significantly reduced major adverse cardiovascular events and mortality in stable atherosclerosis patients—but only in those with on-treatment IL-6 levels below 1.65 ng/L, highlighting IL-6 signaling as a critical mediator of cardiovascular benefit independent of lipid lowering [121].

Supporting this, a large genetic study by Georgakis et al. (358,554 individuals) linked downregulated IL-6 signaling to lower risks of ischemic heart disease, abdominal aortic aneurysm, type 2 diabetes, and better cardiometabolic profiles [122]. However, this genetic profile was also associated with increased risks of neutropenia, infections, atopic conditions, and female infertility, likely due to elevated IL-4 levels—emphasizing the need for careful evaluation of IL-6 receptor blockade as a vascular risk reduction strategy [122].

Many studies have demonstrated that targeting senescent cells provides therapeutic benefits in cardiomyopathies related to aging, ischemia/reperfusion injury, and atherosclerotic vascular disease. This has led to senolytic drugs being considered a promising new class of treatments for cardiovascular disorders. However, recent research has revealed conflicting results and potential adverse effects of senolytic therapies, including exacerbation of cardiac dysfunction, destabilization of atherosclerotic plaques, and increased mortality in animal models, which pose challenges for clinical application [29].

Senolytics are drugs that selectively eliminate senescent cells (SCs), which accumulate with age and contribute to chronic diseases through their SASP. Agents such as dasatinib, quercetin, fisetin, and navitoclax act by transiently inhibiting anti-apoptotic pathways (SCAPs), triggering the clearance of SCs. Since senescent cells reaccumulate slowly, senolytics can be administered intermittently. Preclinical studies have demonstrated their potential to prevent or alleviate multiple age-related diseases [123].

Targeting ECM remodeling represents a promising therapeutic avenue for cardiovascular disease, particularly in conditions characterized by pathological tissue remodeling such as MI. Among these approaches, MMP inhibitors have emerged as potential candidates to attenuate adverse left ventricular (LV) remodeling following MI. In the human heart, exogenous MMP inhibitors may help mitigate structural damage and preserve cardiac function. However, since certain MMPs are essential for physiological tissue repair and homeostasis, therapeutic strategies must aim for the selective inhibition of specific MMP isoforms or localized drug delivery to the affected cardiac tissue [124].

According to the study by Yaras et al., doxycycline abolished diabetes-induced depression in the left ventricular developed pressure and action potential prolongation in diabetic rat hearts [125]. These beneficial effects on mechanical, electrical, and biochemical properties appear to be at least partly due to MMP inhibition, highlighting a role for MMPs in the development of diabetic cardiomyopathy [125]. However, in the multicenter PREMIER trial by Hudson et al., treatment with the selective MMP inhibitor PG-116800 in 253 STEMI patients showed no significant effect on left ventricular remodeling after 90 days compared to placebo, as assessed by changes in LV end-diastolic volume index [126]. According to a study by Tayebjee et al., conducted among 96 patients, circulating pretreatment levels of MMP-9 and TIMP-1 were significantly elevated in individuals with hypertension compared to normotensive controls (*p* = 0.0041 and *p* = 0.0166, respectively). Following treatment, plasma MMP-9 levels significantly decreased, whereas TIMP-1 levels increased (*p* = 0.035 and *p* = 0.005, respectively). MMP-9 levels showed a positive correlation with CHD risk (r = 0.317, *p* = 0.007) and a negative correlation with HDL cholesterol (r = −0.237, *p* = 0.022) but were not associated with cerebrovascular accident (CVA) risk. No significant correlations were observed between TIMP-1 levels and either CHD or CVA risk scores [127]. Despite recent advances, MMPs are associated with potential adverse effects. Dysregulated MMP activity, either through excessive activation or broad inhibition, can paradoxically promote fibrosis and chronic inflammation. In cardiovascular disease, sustained hyperactivity of MMP-2 and MMP-9 drives ECM degradation, maladaptive remodeling and ventricular dysfunction [128]. Currently, the selection of specific MMPs as therapeutic targets remains largely unguided owing to the limited availability of mechanistic and translational data. Further investigations are required to establish strategies for their selective modulation in order to minimize potential off-target effects. Importantly, MMPs constitute only one component of the intricate network governing ECM remodeling, and other elements of the ECM should likewise be considered as potential therapeutic targets [128]. A comprehensive strategy is needed to guide the development, evaluation, and clinical integration of ECM-targeted therapies. This includes early assessment of pharmacokinetics, pharmacodynamics, and safety in diverse populations, including older adults. Continued research is essential to define selective MMP modulation approaches and ensure their safe and effective use throughout the drug lifecycle [129].

RNA-based therapies are increasingly used to treat cardiovascular diseases, especially in hypertension, where several small interfering RNA(siRNA) drugs have shown effectiveness in clinical trials. Beyond siRNAs, other RNA classes are gaining attention. mRNA drugs aimed at promoting revascularization of the infarcted heart are currently under clinical investigation, offering the potential for myocardial protection and regeneration through paracrine factor expression. Emerging approaches involving microRNAs and gene editing are addressing complex cardiovascular conditions in innovative ways. RNA-based gene editing holds promise for permanent cures of monogenic diseases and long-term management of complex disorders like hypertension. Similarly, microRNAs show potential in cardiac muscle regeneration. This review summarizes the current advances in RNA-based therapies for cardiovascular disease [130].

## 8. Conclusions

The global rise in aging-related cardiovascular diseases underscores an urgent need to better understand and therapeutically address the molecular mechanisms driving pathological remodeling of the heart and vasculature. As this review highlights, cardiovascular aging is driven by interrelated processes—including chronic low-grade inflammation (inflammaging), cellular senescence, oxidative stress, and maladaptive ECM remodeling—that collectively disrupt tissue homeostasis and impair cardiovascular function. These mechanisms underlie a spectrum of conditions such as hypertension, atrial fibrillation, atherosclerosis, and heart failure, all of which share common pathological features: endothelial dysfunction, fibrosis, and progressive organ stiffness. Despite major advances in identifying the hallmarks of cardiovascular aging, several critical gaps in knowledge remain. The precise interactions among senescence, mitochondrial dysfunction, and systemic inflammation are still incompletely understood, especially in the context of sex differences and comorbidity-driven variability. Furthermore, while ECM remodeling has emerged as a central determinant of disease progression, the dual role of matrix turnover in both physiological repair and pathological fibrosis poses challenges for therapeutic targeting. Importantly, molecular therapies offer significant promise in modifying these pathological processes. Statins, traditionally used for lipid lowering, exhibit anti-inflammatory and endothelial-protective pleiotropic effects. Agents targeting oxidative stress (e.g., Nrf2 activators), senescence (e.g., senolytics), and inflammatory pathways (e.g., IL-6 and NLRP3 inhibitors) have shown encouraging preclinical results. Similarly, ECM-directed therapies, including selective MMP inhibitors, are under investigation as strategies to prevent or attenuate adverse cardiovascular remodeling. RNA-based therapeutics—including siRNAs, mRNAs, and gene editing technologies—are revolutionizing cardiovascular medicine by enabling the precise targeting of disease-driving genes and molecular pathways. Nevertheless, the long-term efficacy and safety profiles of emerging molecular therapies remain insufficiently characterized, underscoring the need for comprehensive clinical trials and extended follow-up studies. In conclusion, understanding of cardiovascular remodeling provides the foundation for next-generation therapies aimed at halting or even reversing disease progression. In an era of an aging population, therapies that target the molecular basis of cardiovascular remodeling are essential for halting disease progression, improving treatment outcomes, and enhancing patient prognosis.

## Figures and Tables

**Table 1:** Hallmarks of aging [14,15,16].

Hallmark	Meaning
Genomic instability	Accumulation of DNA damage and mutations with age, increasing risk of cancer and neurological diseases
Telomere attrition	Telomere shortening during cell division, leading to cellular senescence
Epigenetic alterations	Age-related changes in gene expression not involving DNA sequence, contributing to disease
Loss of proteostasis	Decline in the cell’s ability to fold and clear proteins, promoting aging
Dysregulated nutrient sensing	Impaired response to nutrient levels, linked to metabolic disorders (e.g., diabetes)
Mitochondrial dysfunction	Reduced mitochondrial efficiency, causing energy loss and oxidative stress
Cellular senescence	Permanent cell cycle arrest, driving aging processes
Stem cell exhaustion	Decrease in stem cell number and function, limiting tissue repair
Altered intercellular communication	Disruption of hormonal and cellular signaling, affecting tissue function
Compromised autophagy	Impaired clearance of damaged proteins, linked to neurodegeneration and immune aging
Microbiome disturbances	Loss of gut microbial diversity, contributing to inflammation
Splicing dysregulation	Misregulation of RNA splicing, fostering cellular aging
Chronic low-level inflammation (inflammaging)	Persistent mild inflammation, increasing disease risk in the elderly
Mechanical properties alterations	Stiffening and cross-linking of tissues (e.g., collagen), leading to conditions like hypertension

**Table 2:** Overview of senescence-associated secretory phenotype (SASP) components and effects in cardiovascular cell types [28,29,30,31,32,33,34,35,36,37,38,39,40,41,42,43,44,45,46,47,48,49,50,51,52,53,54,55,56,57,58,59,60,61,62,63].

General features	SENESCENCE-ASSOCIATED SECRETORY PHENOTYPE (SASP) is a heterogeneous collection of secreted factors produced by senescent cells: · **pro-inflammatory cytokines:** IL-1α/β, IL-6, TNF-α, IL-8 · **fibrotic and pro-hypertrophic factors:** TGF-β, activin A, GDF15, CCN1 · **chemokines:** CCL2/MCP-1, CXCL1, CXCL8, attracting monocytes, neutrophils, T and B cells · **damage-associated molecular patterns (DAMPs)** · **proteolytic enzymes:** MMPs, elastases, cathepsins · **hemostatic and vasoactive mediators:** PAI-1, PAI-2, prostanoids, bradykinins, endothelin-1				
Cell- type specific aspects	**Senescent cardiomyocytes**	**Senescent fibroblast**	**Senescent endothelial cells**	**Senescent VSMCs**	**Immune cells**
	IL-1, IL-6, TNF-α, TGF-β, GDF15	IL-1, IL-6, TNF-α, TGF-β, CCN1.	Cytokines + reduced angiogenic factors.	IL-1β, IL-6, TNF-α, MMPs.	Macrophages, T cells neutrophils, mast cells, NK cells.
	DDR activation, ER stress, mitochondrial dysfunction, elevated ROS levels contractile abnormalities, epigenetic dysregulation (miRNAs), promotes fibroblast activation: inflammation, fibrosis, hypertrophy.	Central role in ECM remodeling; senescent by p53/p-21, drive paracrine hypertrophy, fibrosis.	NO, EDHF, mitochondrial dysfunction, RAAS activation, oxidative stress, impaired angiogenesis, promote vascular inflammation.	Telomere attrition, DNA damage, oxidative stress, defective autophagy; induce immune recruitment, matrix degradation, osteogenic shift: vascular stiffening, calcification.	Respond to SASP signals, further shaping inflammation, senescence propagation, or clearance.
Functional roles in CVS	**Acute** → immune cell recruitment, clearance of senescent cells, wound healing, regeneration. **Chronic** → Persistent inflammation, impaired stem cell function, fibrosis, hypertrophy, ECM degradation, vascular dysfunction and progression of age-related cardiac diseases: HFpEF, atherosclerosis, atrial fibrillation, diabetic cardiomyopathy.				

## Data Availability

The data used in this article are sourced from materials mentioned in the References section.

## Abbreviations

The following abbreviations are used in this manuscript:

DALYs	Disability-adjusted life years
IHD	Ischemic Heart Disease
COPD	Chronic obstructive pulmonary disease
CVD	Cardiovascular disease
AHA	American Heart Association
TNF-α	Tumor necrosis factor alpha
IL-1β	Interleukin 1 beta
CRP	C-reactive protein
LPS	Lipopolysaccharide
TLRs	Toll-like receptors
NLRP3	Nod-like receptor pyrin domain-containing 3
HFpEF	Heart failure with preserved ejection fraction
ROS	Reactive oxygen species
mtDNA	Mitochondrial DNA
DAMP	Damage-associated molecular pattern
NO	Nitric Oxide
hsCRP	High-sensitivity C-reactive protein
AMPK	5′ AMP-activated protein kinas
Nrf2	Nuclear factor erythroid 2–related factor 2
SASP	Senescence-associated secretory phenotype
DDR	DNA damage response
ER	Endoplasmic reticulum
VSMCs	Vascular smooth muscle cells
RAAS	Renin–angiotensin–aldosterone system
ECM	The extracellular matrix
MMPs	Matrix metalloproteinases
LOX	Lysyl oxidase
LOXL	LOX-like
EMT	Epithelial-mesenchymal transition
IPF	Idiopathic pulmonary fibrosis
TIMPs	Tissue inhibitors of metalloproteinases
MLR	Media-to-Lumen Ratio
WLR	Wall-to-Lumen Ratio
VEGF	Vascular Endothelial Growth Factor
AF	Atrial Fibrillation
OR	Odds Ratio
PFDR	P-value adjusted for False Discovety Rate
HR	Hazard Ratio
TCFA	Thin-Cap Fibroatheroma
HF	Heart failure
TGF- β	Transforming Growth Factor Beta
HFrEF	Heart Failure with Reduced Ejection Fraction
RNA-seq	RNA sequencing
ACLY	ATP-Citrate Lyase
LV	Left Ventricle
MI	Myocardial Infarction
CHD	Coronary heart Disease
CKD	Chronic Kidney Disease
eNOS	Endothelial Nitric Oxide Synthase
NF-κB	Nuclear Factor kappa-light-chain-enhancer of activated B cells
SC	Senescent Cells
SCAPs	Senescent Cell Anti-Apoptotic Pathways
STEMI	ST-segment Elevation Myocardial Infarction
siRNA	Small Interfering RNA
mRNA	Messenger RNA
